# Substrate Selectivities of GH78 α-L-Rhamnosidases from Human Gut Bacteria on Dietary Flavonoid Glycosides

**DOI:** 10.3390/molecules30050980

**Published:** 2025-02-20

**Authors:** Bin-Chun Li, Bingbing Wu, Xueting Hou, Guo-Bin Ding

**Affiliations:** 1 Institute of Biotechnology, Key Laboratory of Chemical Biology and Molecular Engineering of Ministry of Education, Shanxi University, Taiyuan 030006, China; wubingbing09@163.com (B.W.); houxueting_sxu@163.com (X.H.); 2 Shanxi Key Laboratory of Biotechnology, Shanxi University, Taiyuan 030006, China; 3 Institutes of Biomedical Sciences/School of Life Sciences, Inner Mongolia University, Hohhot 010070, China

**Keywords:** substrate selectivity, α-L-rhamnosidase, dietary flavonoid glycosides, human gut bacteria

## Abstract

α-L-rhamnosidases play a key role in the metabolism and biodegradation of dietary flavonoid glycosides. We have developed a novel microplate spectrophotometric method to rapidly evaluate the conversion rates and substrate selectivities of mesophilic α-L-rhamnosidases towards citrus flavanone diglycosides by combining with a high-active and thermophilic β-D-glucosidase based on UV-visible spectral differences between citrus flavanone diglycosides and the corresponding aglycones under alkaline conditions. Furthermore, catalytic activities and enzyme kinetics of four α-L-rhamnosidases from human gut bacteria on various dietary flavonoid glycosides with different glycosidic bonds from various subclasses have been explored by HPLC. The α-L-rhamnosidase BtRha78A specifically removed the rhamnose group from the flavones, flavanones and flavonols diglycosides with the α-1,6 glycosidic bonds. Moreover, BtRha78A displayed higher catalytic activities on the rutinose group at 7-OH of the aglycones than at 3-OH. HFM-RhaA preferred to catalyze the flavones, flavanones and dihydrochalcones diglycosides with the α-1,2 glycosidic linkages at the 7-OH. However, this enzyme also showed high catalytic activity on the flavonol diglycoside rutin with the α-1,6 glycosidic bonds at the 3-OH. HFM-RhaC exhibited certain hydrolytic abilities towards all flavonoid diglycosides, and displayed higher activities on the flavonoid diglycosides with the α-1,6 glycosidic bonds. HFM-Rha78 weakly hydrolyzed the flavones, flavanones and dihydrochalcones diglycosides with the α-1,2 glycosidic bonds, and the flavonols diglycosides with α-1,6 glycosidic bonds. All four α-L-rhamnosidases from human gut bacteria did not exhibit catalytic activity towards the flavonoid glycosides with the α-1 glycosidic bonds. It was revealed that the α-L-rhamnosidases from human gut bacteria possessed diverse substrate selectivity on dietary flavonoid diglycosides. The structural basis for the specificity of BtRha78A on the flavonoid diglycosides with α-1,6 glycosidic bonds and the preference of HFM-RhaA on the flavonoid diglycosides with α-1,2 glycosidic bonds have been analyzed by molecular docking.

## 1. Introduction

α-L-Rhamnosidases (EC 3.2.1.40) specifically cleave the terminal α-L-rhamnose group from plants natural glycosides including flavonoids glycosides and ginsenosides [1]. α-L-Rhamnosidases have widely occurred in nature and been found in animals, plants and microorganisms. α-L-Rhamnosidases have been classified into glycoside hydrolase families GH28, GH78 and GH106. The cloned and characterized α-L-rhamnosidases have been mainly distributed in the family GH78. To date, eight structures from GH78 family α-L-rhamnosidases (Rha78s) have been resolved: the α-L-rhamnosidase BsRhaB from *Bacillus* sp. GL1 (PDB id, 2OKX) [2], the α-L-rhamnosidase BtRha78A from *Bacteroides thetaiotaomicron* VPI-5482 (BT1001, PDB id, 3CIH) [3], the α-L-rhamnosidase DtRha from *Dictyoglomus thermophilum* H-6-12 (PDB id, 6I60) [4], the α-L-rhamnosidase KoRha from *Klebsiella michiganensis* KCTC 1686 (PDB id, 4XHC) [5], the α-L-rhamnosidase SaRha78A from *Streptomyces avermitilis* (PDB id, 3W5N) [6], the α-L-rhamnosidase N12-Rha from *Aspergillus niger* TS528 (PDB id, 8IWF) [7], the α-L-rhamnosidase AoRhaA from *Aspergillus oryzae* RIB40 (PDB id, 8HMM) [8], and the α-L-rhamnosidase AtRha from *Aspergillus terreus* CCF 3059 (PDB id, 6GSZ) [9]. α-L-Rhamnosidases have turned out to be a biotechnologically important enzyme for biotransformation of natural flavonoid diglycosides into the corresponding flavonoid glucosides with higher bioactivities and better bioavailability. The flavonoids glucosides isoquercitrin, hesperetin-7-O-glucoside, and prunin with higher bioactivities and better bioavailability have been produced from natural flavonoids diglycosides by using the α-L-rhamnosidases or naringinase [10,11,12,13]. In the food industry, the α-L-rhamnosidases have been used for the debittering of citrus fruit juices and the improvement of wine aroma [14,15].

At present, the methods for measuring catalytic activities of the α-L-rhamnosidases contained high performance liquid chromatography (HPLC) [16], thin-layer chromatography (TLC) [17], and UV-visible spectrophotometry by using a synthetic substrate *p*-nitrophenyl-α-L-rhamnopyranoside (*p*NPR) [18]. Although HPLC is accurate in quantification, it has a slow detection speed. The TLC boasts low cost and simple operation. However, it fails to quantify precisely. The substrate *p*NPR is suitable for rapidly detecting catalytic activity and high-throughput screening of the α-L-rhamnosidases; however, it is costly and has a significant structural disparity from natural flavonoid glycosides and cannot genuinely reflect catalytic activity of the α-L-rhamnosidases on natural substrates.

**Figure 1:**
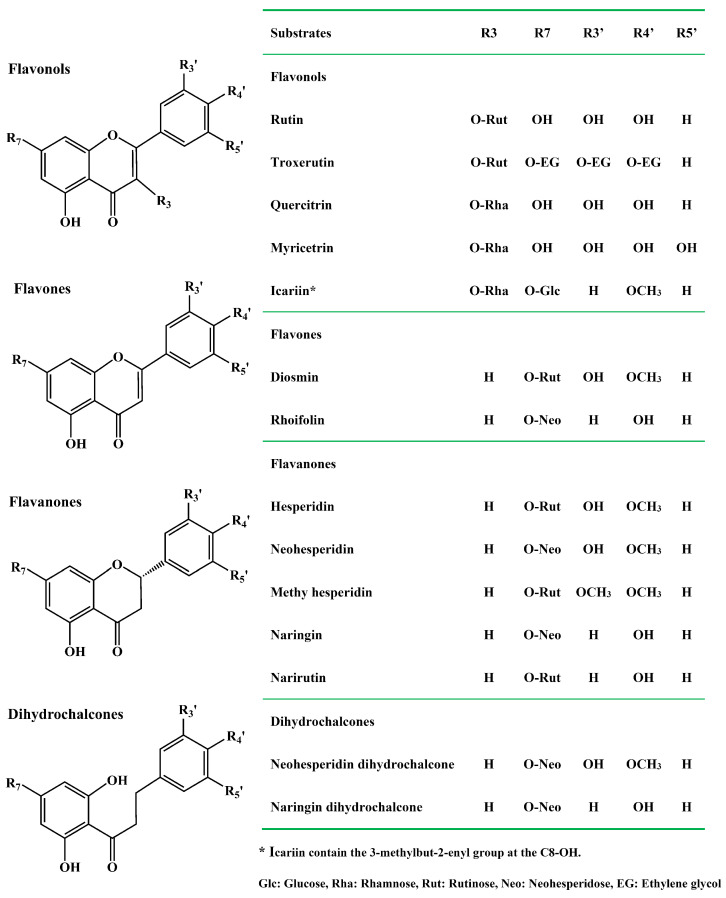
Molecular structures and subclasses of common dietary flavonoid glycosides.

Flavonoids are plant polyphenolic chemicals with diverse structures. According to molecular structures, flavonoids could be divided into six subgroups: flavanones, flavones, flavanols, flavonols, isoflavones, and anthocyanidins [19]. The flavonoids have multiple therapeutic benefits and biological activities including anti-oxidation [20], anti-inflammatory [21,22], anticancer [23], antiviral [24], antidiabetic [25], neuroprotection [26,27], cardiovascular protection [28]. Plants, fruits and herb-medicines are rich source of dietary flavonoids. Natural flavonoids mainly exist in the form of glycosides especially the diglycosides such as rutin, hesperidin, neohesperidin, naringin, narirutin, diosmin, naringin dihydrochalcone, and neohesperidin dihydrochalcone (Figure 1). The disaccharide moiety α-L-rhamnosyl-β-D-glucoside attached to the flavonoid aglycones at the position 7-OH or 3-OH with a bitter taste. The disaccharide group mainly exists in two forms. Naringin, neohesperidin and rhoifolin contain the neohesperidose, where α-L-rhamnose and β-D-glucose are linked by an α-1,2 glycosidic bond. However, hesperidin, narirutin and rutin include the rutinose, with α-L-rhamnose and β-D-glucose linked the α-1,6 glycosidic bond. Additionally, the flavonoid rhamnosides are produced by transferring the rhamnose group into the aglycones at the position 3-OH, which is called α-1 glycosidic bond including myricetin, quercitrin and icariin. However, natural flavonoid diglycosides display poor water solubility, low bioavailability and bioactivity. Enzymatic modification of natural flavonoid diglycosides by the α-L-rhamnosidase generated the corresponding flavonoid glucosides with better bioavailability and higher biological function [29,30,31,32,33].

Owing to the differences in the substrate-binding pockets in the α-L-rhamnosidases and molecular structures of various flavonoid glycosides, certain α-L-rhamnosidases exhibit a preference for hydrolyzing the α-1,2 glycosidic bond or α-1,6 glycosidic bond in natural flavonoid diglycosides. Therefore, different α-L-rhamnosidases exhibited diverse substrate selectivities (Table 1). Exploring the substrate selectivities of the α-L-rhamnosidases is helpful for selecting a suitable candidate for the preparation of high-value flavonoid glucosides or aglycones. The α-L-rhamnosidases from human intestinal bacteria play a key role in the biodegradation and metabolism of dietary flavonoid glycosides with diverse sources and complex molecular structures into the glucosides and aglycones. The flavonoid glucosides and aglycones exerting higher biological function and better bioavailability can then be absorbed and utilized by the human body. The diversity of human gut bacteria varies greatly among different individuals, and the number and enzymatic properties of the α-L-rhamnosidases from human gut bacteria in different individuals also vary greatly. Therefore, elucidating structural basis for substrate selectivity of the α-L-rhamnosidases from human gut bacteria on dietary flavonoid glycosides is crucial for understanding how human gut bacteria metabolize dietary flavonoid glycosides.

Firstly, to establish a spectrophotometric method for rapid evaluation of substrate selectivities of mesophilic α-L-rhamnosidases on citrus flavanone diglycosides in 96-well microplate by microplate reader, spectral properties of four citrus flavanone diglycosides hesperidin, neohesperidin, naringin, and narirutin and their corresponding aglycones hesperetin and naringenin under different conditions were carried out. After mesophilic α-L-rhamnosidases hydrolyzing citrus flavanone diglycosides into flavanone glucosides by cleaving the terminal α-rhamnosyl group at moderate temperatures in the microplate, a high-active and thermophilic β-D-glucosidase was applied for hydrolyzing the generated flavanone glucosides to produce the corresponding aglycones. The final aglycones produced could be quantified by using a microplate reader. Substrate selectivities of these four α-L-rhamnosidases on citrus flavonoid diglycosides containing different glycosidic linkages were confirmed by the method HPLC. The catalytic activities and enzyme kinetics of the α-L-rhamnosidases on dietary flavonoid glycosides have been measured for exploring the substrate selectivities of the α-L-rhamnosidases from human gut bacteria on dietary flavonoid glycosides. The interactions of key residues of the α-L-rhamnosidases from human gut bacteria with dietary flavonoid glycosides were analyzed by molecular docking.

## 2. Results

### 2.1. Spectroscopic Characterization of Citrus Flavanone Diglycosides and Aglycones

**Figure 2:**
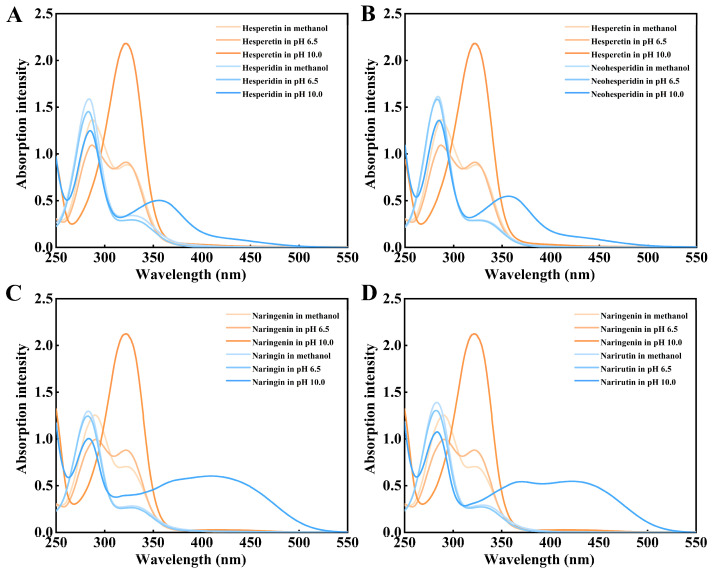
UV-visible spectra of citrus flavanone diglycosides and the corresponding aglycones under different conditions. (**A**). UV-visible spectra of hesperidin and hesperetin. (**B**). UV-visible spectra of neohesperidin and hesperetin. (**C**). UV-visible spectra of naringin and naringenin. (**D**). UV-visible spectra of narirutin and naringenin.

The main difference between the molecular structures (Figure 1) of four citrus flavanone diglycosides is that hesperidin and narirutin contain the α-1,6 glycosidic bond but neohesperidin and naringin contain the α-1,2 glycosidic bond. UV-visible spectra of citrus flavanone diglycosides hesperidin, neohesperidin, naringin, and narirutin and their corresponding aglycones hespederin and naringenin at different conditions were performed by full wavelength scanning. The maximum absorption peak of all four citrus flavanone diglycosides appeared at 283 nm in the methanol, acidic pH 6.5, and alkaline pH 10.0 (Figure 2). There were no significant spectral differences between citrus flavanone diglycosides and their aglycones in the methanol and acidic pH 6.5, although the aglycones had higher absorption values than the diglycosides at the wavelengths of greater than 300 nm (Figure 2). However, in pH 10.0, the maximum absorption peak of the aglycones hesperetin and naringenin occurred at 320 nm (Figure 2), which might due to the autoxidation in alkaline conditions [44]. The new maximum absorption peak of the aglycones hesperetin and naringenin at 320 nm in alkaline pH 10.0 could be considered as their characteristic peak, because citrus flavanone diglycosides exhibited a very low absorption at 320 nm. Standard curves for quantification of citrus flavanone diglycosides and their corresponding aglycones were generated by the microplate and microplate reader (Figure S1).

### 2.2. Microplate Spectroscopic Method for Rapid Evaluation of Substrate Selectivity of Mesophilic α-L-Rhamnosidases on Citrus Flavanone Diglycosides

**Figure 3:**
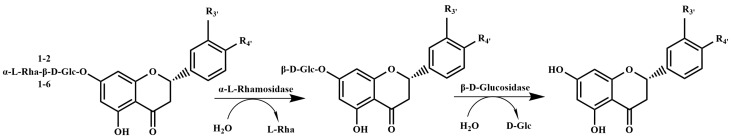
Bioconversion pathway of citrus flavanone diglycosides into the corresponding aglycone by combining the α-L-rhamnosidase with the β-D-glucosidase.

α-L-Rhamnosidases might hydrolyze citrus flavanone diglycosides into the corresponding flavanone glucosides. Nevertheless, the generated flavanone glucosides will be completely converted into the aglycones, if β-D-glucosidase activity is present along with the α-L-rhamnosidase (Figure 3). A high-active double mutant N221S/P3421L (described as TnBgl1A-DM) of thermophilic β-D-glucosidase TnBgl1A from *Thermotoga neapolitana* was used for efficiently hydrolyzing the citrus flavanone glucosides into the corresponding aglycones [45]. On the one hand, high-active β-D-glucosidase can efficiently hydrolyze citrus flavanone glucosides and the generated glucosides can be completely converted into the aglycones. On the other hand, the reason for choosing a thermophilic β-D-glucosidase is that mesophilic α-L-rhamnosidases are rapidly thermally inactivated when thermophilic β-D-glucosidase catalyzes at high temperature, so that the reaction will not continue to produce the glucosides and affect the quantification of the citrus flavanone aglycones. Therefore, the relative concentration of citrus flavanone aglycones detected by a microplate reader might be used for rapidly evaluating enzymatic activities and conversion rates of mesophilic α-L-rhamnosidases on citrus flavanone diglycosides by combining with a high-active and thermophilic β-D-glucosidase.

**Figure 4:**
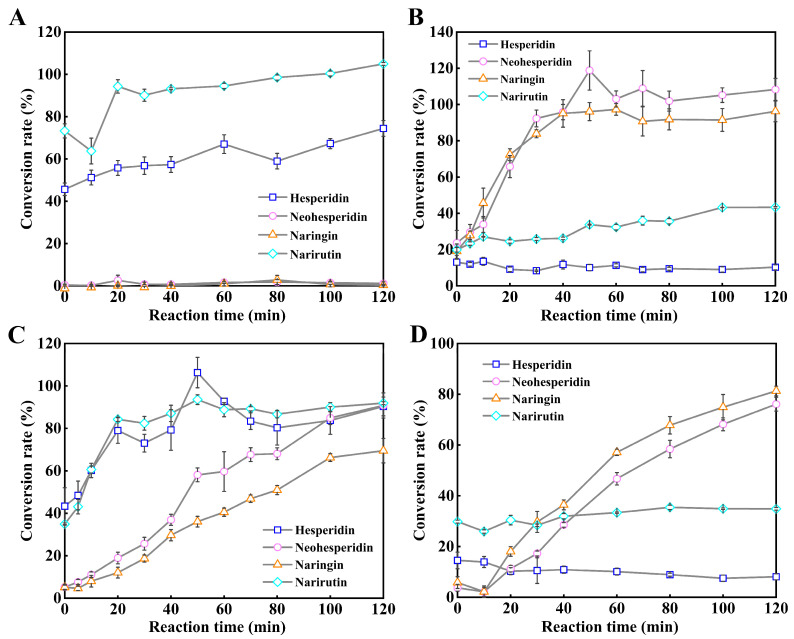
Substrate selectivities of four mesophilic α-L-rhamnosidases on four citrus flavanone diglycosides by using the UV-visible spectrophotometry in the microplate. (**A**). Conversion rates of BtRha78A on citrus flavanone diglycosides with varied reaction times. (**B**). Conversion rates of HFM-RhaA on citrus flavanone diglycosides with varied reaction times. (**C**). Conversion rates of HFM-RhaC on citrus flavanone diglycosides with varied reaction times. (**D**). Conversion rates of HFM-Rha78 on citrus flavanone diglycosides with varied reaction times. All reactions were performed in triplicate, and error bars represent the standard deviations of mean.

In order to verify the feasibility of the spectroscopic method based on hydrolyzing in the 96-well microplate and monitoring by a microplate reader for rapid evaluation of conversion rates and substrate selectivities (α-1,6 or α-1,2) of mesophilic α-L-rhamnosidases on citrus flavanone diglycosides, four α-L-rhamnosidases from human gut bacteria including three Rha78s from human fecal metagenome and one Rha78s from *B. thetaiotaomicron* VPI-5482 were chosen as the models. Four α-L-rhamnosidases and the β-D-glucosidase TnBgl1A-DM have been over-expressed in *E. coli* BL21(DE3) and purified into homogeneity by the analysis of SDS-PAGE (Figures S2 and S3). The conversion rates of four mesophilic α-L-rhamnosidases on four citrus flavanone diglycosides with varied reaction times have been monitored by using the microplate UV-visible method in order to rapidly evaluate the substrate selectivities (α-1,6 or α-1,2) of these α-L-rhamnosidases on citrus flavanone diglycosides (Figure 4).

It was indicated that narirutin and hesperidin were unstable and autohydrolyzed at the temperature 80 °C (Figure 4). The high-active and thermophilic β-D-glucosidase TnBgl1A-DM displayed no activity against citrus flavanone diglycosides and mesophilic α-L-rhamnosidases quickly were inactive at the temperature 80 °C (Figure 4). The α-L-rhamnosidase BtRha78A displayed high hydrolytic activities on narirutin and hesperidin containing the rutinoside with the α-1,6 glycosidic bonds, and the conversion rates of BtRha78A on narirutin and hesperidin were 90.1% and 56.8% in 30 min at 37 °C, respectively (Figure 4A). However, BtRha78A hardly catalyzed the neohesperidin and naringin with the α-1,2 glycosidic bonds (Figure 4A). It was indicated that the α-L-rhamnosidase BtRha78A specifically hydrolyzed the citrus flavanone diglycosides with the α-1,6 glycosidic bonds. The α-L-rhamnosidase HFM-RhaA possessed high conversion rates on neohesperidin and naringin, and the conversion rates of HFM-RhaA on neohesperidin and naringin were 92.3% and 83.9% in 30 min at 37 °C, respectively (Figure 4B). HFM-RhaA preferred the hydrolysis of the citrus flavanone diglycosides with the α-1,2 glycosidic bonds. The conversion rates of the α-L-rhamnosidase HFM-RhaC on narirutin and hesperidin were 82.4% and 73.0% in 30 min at 37 °C, respectively (Figure 4C), and the conversion rates of HFM-RhaC on neohesperidin and naringin were 90.8% and 69.5% in 120 min at 37 °C, respectively (Figure 4C). The α-L-rhamnosidase HFM-RhaC was capable of hydrolyzing all four citrus flavanone diglycosides and displayed a preference toward the α-1,6 glycosidic bonds. The α-L-rhamnosidase HFM-Rha78 displayed moderate catalytic activities on neohesperidin and naringin, and the conversion rates of HFM-Rha78 on neohesperidin and naringin were 86.6% and 87.5% in 120 min at 37 °C, respectively (Figure 4D). The α-L-rhamnosidase HFM-Rha78 preferred to hydrolyze the citrus flavanone diglycosides with the α-1,2 glycosidic bonds.

**Figure 5:**
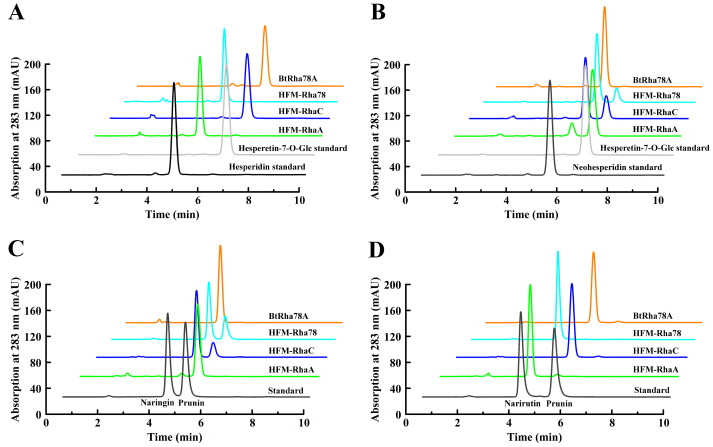
HPLC chromatograms for biotransformation of four citrus flavanone diglycosides by four mesophilic α-L-rhamnosidases. (**A**). HPLC chromatograms for biotransformation of mesophilic α-L-rhamnosidases on 1 mM hesperidin for 30 min at 37 °C in pH 6.0 (HFM-RhaA and HFM-RhaC) or pH 6.5 (BtRha78A and HFM-Rha78) with 0.1 mg mL^−1^ of the enzymes. (**B**). HPLC chromatograms for biotransformation of mesophilic α-L-rhamnosidases on 1 mM neohesperidin for 30 min at 37 °C in pH 6.0 (HFM-RhaA and HFM-RhaC) or pH 6.5 (BtRha78A and HFM-Rha78) with 0.1 mg mL^−1^ of the enzymes. (**C**). HPLC chromatograms for biotransformation of mesophilic α-L-rhamnosidases on 1 mM naringin for 30 min at 37 °C in pH 6.0 (HFM-RhaA and HFM-RhaC) or pH 6.5 (BtRha78A and HFM-Rha78) with 0.1 mg mL^−1^ of the enzymes. (**D**). HPLC chromatograms for biotransformation of mesophilic α-L-rhamnosidases on 1 mM narirutin for 30 min at 37 °C in pH 6.0 (HFM-RhaA and HFM-RhaC) or pH 6.5 (BtRha78A and HFM-Rha78) with 0.1 mg mL^−1^ of the enzymes.

The feasibility of this novel microplate UV-visible approach was confirmed by monitoring hydrolytic capabilities of four α-L-rhamnosidases from human gut bacteria towards four citrus flavanone diglycosides by HPLC. The HPLC chromatograms exhibited that the α-L-rhamnosidases BtRha78A and HFM-RhaC effectively hydrolyzed hesperidin and narirutin into hesperetin-7-O-glucoside and prunin, respectively (Figure 5A,D). The α-L-rhamnosidase HFM-RhaA highly catalyzed neohesperidin and naringin into hesperetin-7-O-glucoside and prunin, respectively, and HFM-RhaC and HFM-Rha78 moderately converted neohesperidin and naringin into hesperetin-7-O-glucoside and prunin, respectively (Figure 5B,C). The HPLC results were in accordance with those results from the microplate UV-visible method. It was suggested that the novel microplate UV-visible method might be applied for rapid evaluation of substrate selectivities (α-1,6 or α-1,2) of mesophilic α-L-rhamnosidase towards citrus flavonoid diglycosides.

### 2.3. Substrate Selectivity of α-L-Rhamnosidases from Human Gut Bacteria on Dietary Flavonoid Diglycosides by HPLC

In order to systematically explore the substrate selectivities of the α-L-rhamnosidases from human gut bacteria on dietary flavonoid glycosides, specific activities and enzyme kinetics of four α-L-rhamnosidases from human gut bacteria on a wide variety of flavonoid glycosides (Figure 1) including different flavonoid subclasses, the glycosylation at different positions of the aglycones, and different glycosidic bonds were determined by HPLC. Standard curves for quantification of flavonoid glycosides or their corresponding aglycones were performed by HPLC (Figure S4). BtRha78A specifically removed the rhamnose group from the flavones, flavanones, and flavonols diglycosides with the α-1,6 glycosidic bonds (Table 2). Moreover, BtRha78A displayed higher catalytic activities on the flavone diglycoside diosmin and the flavanone diglycosides narirutin and hesperidin introducing the rutinose groups at 7-OH of the aglycones than the flavonol diglycosides rutin and troxerutin carrying the rutinose groups at 3-OH (Table 2). However, BtRha78A could scarcely hydrolyze the α-1,2 glycosidic bond (Table 2). HFM-RhaA preferred to catalyze the flavones, flavanones, and dihydrochalcones diglycosides containing α-1,2 glycosidic linkages at the 7-OH of the aglycones. However, this enzyme also showed high catalytic activity on the flavonol diglycoside rutin with the α-1,6 glycosidic bond at 3-OH (Table 2). HFM-RhaC exhibited certain hydrolytic abilities towards all flavonoid diglycosides, and displayed higher catalytic activities on the flavones and flavanones diglycosides with the α-1,6 glycosidic bond than those flavonoid diglycosides with the α-1,2 glycosidic bonds (Table 2). HFM-Rha78 weakly hydrolyzed the flavones, flavanones and dihydrochalcones diglycosides with the α-1,2 glycosidic bonds, and the flavonols diglycosides with α-1,6 glycosidic bonds. It was noteworthy that all four α-L-rhamnosidases from human gut bacteria could not catalyze the hydrolysis of the flavonol glycosides with the α-1 glycosidic bond, such as quercitrin, myricetrin and icariin (Table 2).

Michaelis−Menten kinetic parameters of four α-L-rhamnosidases from human gut bacteria on four dietary flavonoids diglycosides have been determined (Table 3). The *k*_cat_/*K*_M_ values of BtRha78A on rutin and hesperidin were 199.5 s^−1^ M^−1^ and 3138.8 s^−1^ M^−1^, respectively, indicating higher catalytic efficiency on hesperidin than rutin. The catalytic efficiency (*k*_cat_/*K*_M_) of HFM-RhaA on neohesperidin and naringin was 10,154 s^−1^ M^−1^ and 13,512 s^−1^ M^−1^, respectively; yet, this enzyme had higher affinity on neohesperidin than naringin. The *k*_cat_/*K*_M_ values of HFM-RhaC on rutin, hesperidin, neohesperidin and naringin were 457.9 s^−1^ M^−1^, 27,025 s^−1^ M^−1^, 1025.8 s^−1^ M^−1^, 654.2 s^−1^ M^−1^, respectively. This enzyme possessed highest catalytic efficiency on hesperidin. HFM-Rha78 had weak catalytic efficiency on neohesperidin and naringin.

### 2.4. Structral Basis for Substrtae Selectivity of α-L-Rhamnosidases from Human Gut Bacteria on Dietary Flavonoid Glycosides

**Figure 6:**
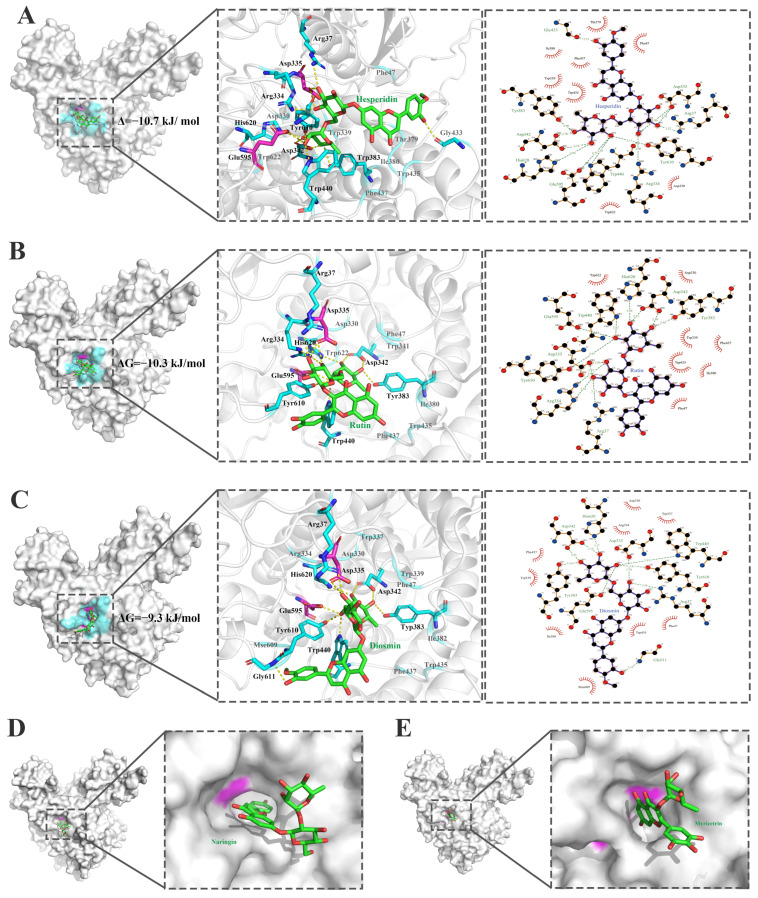
Interactions of the substrate-binding pocket of the α-L-rhamnosidase BtRha78A with five flavonoid glycosides. (**A**). Hesperidin. (**B**). Rutin (**C**). Diosmin. (**D**). Naringin. (**E**). Myricetrin. The flavonoid glycosides are displayed in green ball-and-sticks. Key residues of BtRha78A are highlighted. Catalytic residues are colored in magenta. The residues which are involved in hydrogen bonds and π–π stacking interactions are shown in cyan. The hydrogen bond is represented in yellow dot line. The interactions were analyzed by using the softwares PyMOL education 3.0.5 and LigPlot^+^ 1.4.

The α-L-rhamnosidases from human gut bacteria exhibited diverse substrate selectivities on dietary flavonoid glycosides. Structural basis for substrate selectivities of the α-L-rhamnosidases from human gut bacteria on dietary flavonoid glycosides has been analyzed by the computer-aided tools. The 3D structure for the complex of the α-L-rhamnosidase BtRha78A from *B. thetaiotaomicron* VPI-5482 with the ligand Tris (L-rhamnose analogue) has been resolved (PDB id, 3CIH) [3]. The interactions (Figure 6) of the residues from substrate-binding pocket of BtRha78A with the flavonoid glycosides hesperidin, rutin, diosmin, naringin, and myricetrin by molecular docking revealed that BtRha78A exhibits the specificity on flavonoid diglycosides with the α-1,6 glycosidic bonds. The residues Arg37, Asp335, Asp342, Tyr383, Trp440, Glu595, Tyr610, and His620 in the substrate-binding pocket formed the hydrogen bonds with hesperidin, rutin and diosmin. The general acid Asp335 and general base Glu595 participated in the hydrogen bonds with the rutin, hesperidin and diosimin. Among them, BtRha78A have the lowest binding energy with hesperidin and the greatest number of hydrogen bonds. The aromatic residues Phe47, Trp435, Phe437 and Trp622 produced π–π stacking interactions with the pyranose rings of the sugar group. However, the naringin substrates containing the α-1,2 glycosidic bond and myricetin with the α-1 glycosidic bond could be docked into the substrate-binding pocket. This different positioning of two saccharides could affect substrate binding at the catalytic pocket. In addition, the experiment proved that BtRha78A had the best catalytic activity on hesperidin, while no catalytic activity was detected on naringin and myricetin.

**Figure 7:**
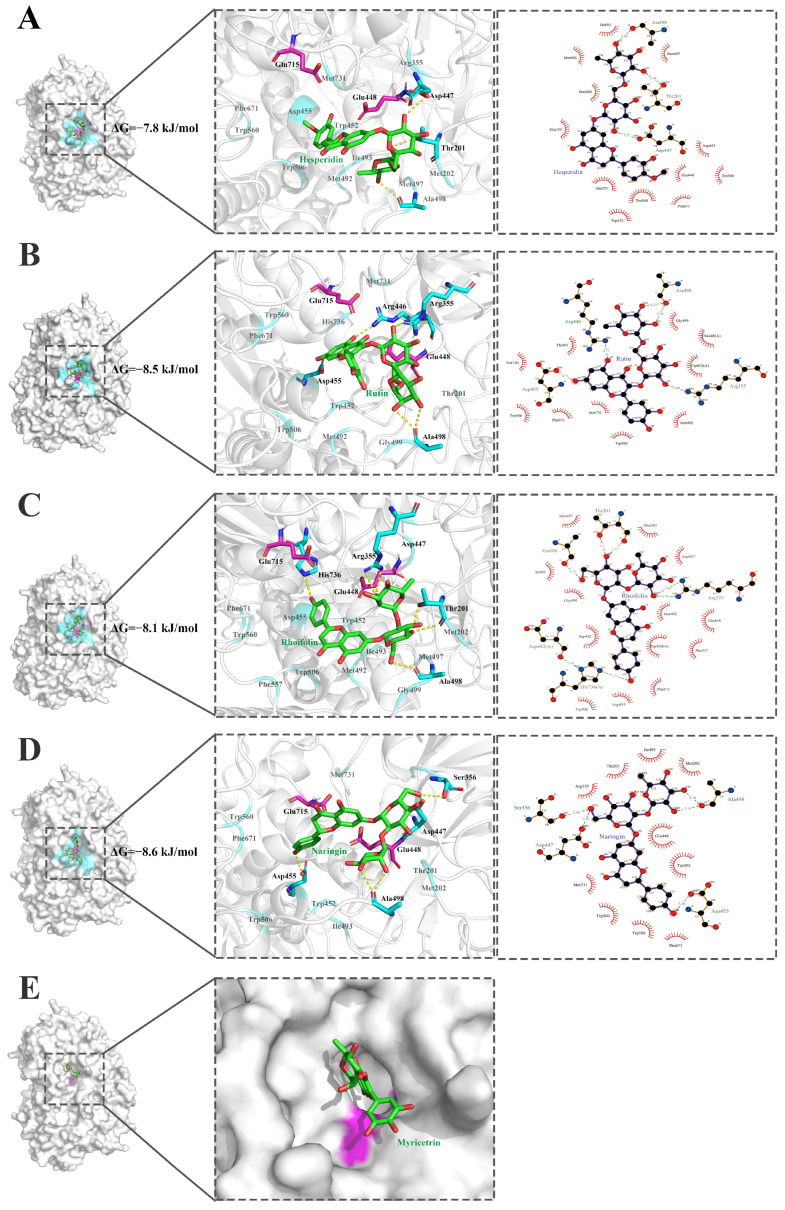
Interaction of the substrate-binding pocket of the α-L-rhamnosidase HFM-RhaA with five flavonoid glycosides. (**A**). Hesperidin. (**B**). Rutin (**C**). Rhoifolin. (**D**). Naringin. (**E**). Myricetrin. The flavonoid glycosides are displayed in green ball-and-sticks. Key residues of HFM-RhaA are highlighted. The catalytic residues are colored in magenta. The residues which are involved in hydrogen bonds and π–π stacking interactions are shown in cyan. The hydrogen bond is represented in yellow dot line. The interactions were analyzed by using the softwares PyMOL education 3.0.5 and LigPlot^+^ 1.4.

Since no crystal structure of HFM-RhaA has been available yet, the 3D model of HFM-RhaA has been predicted by AlphaFold2 and submitted into the database UniProt. Structural analysis for the complex of HFM-RhaA with five flavonoid glycosides naringin, rhoifolin, rutin, hesperidin, and myricetrin were employed by molecular docking. The residue Ala498 might be the key residue for catalytic activity of HFM-RhaA on the flavonoid diglycosides, because the residue Ala498 formed hydrogen bonds with all the flavonoid diglycosides naringin, rhoifolin, rutin and hesperidin (Figure 7). The residues Glu448, Trp452, Trp506 and Phe671 formed hydrophobic interactions with catalytically active flavonoid diglycosides and might regulate hydrolytic activities of HFM-RhaA on these substrates (Figure 7). HFM-RhaA produced more hydrogen bonds with the flavonoid diglycosides naringin and rhoifolin containing the α-1,2 glycosidic bond than the substrates with the α-1,6 glycosidic bond, and HFM-RhaA preferred to catalyze the flavonoids diglycosides containing the α-1,2 glycosidic linkages. For the flavonoid diglycosides rutin and hesperidin with the α-1,6 glycosidic bond, the binding energy of HFM-RhaA with rutin was lower and the number of hydrogen bonds was more than hesperidin, and thus this enzyme showed higher catalytic activity on rutin than hesperidin. The myricetin could not be docked into HFM-RhaA, and thus HFM-RhaA could not catalyze the removal of α-rhamnosyl group from myricetin (Figure 7).

## 3. Discussion

Citrus flavanone aglycones presented the characteristic absorption peak at 320 nm under alkaline pH 10.0. This is consistent with previous study about hesperidin and hesperetin [46]. Based on the spectral difference of hesperidin with hesperetin, the hesperidin in citrus-based foods could be quantified by monitoring the aglycone hesperetin by using a fungal diglycosidase. Compared with the difference in extinction coefficients (∆ε_320_) between rutin and its aglycone quercetin [47], the difference in extinction coefficients (∆ε_320_) (Figure 2) between citrus flavanone diglycosides and their aglycones was more suitable for quantifying the citrus flavanone aglycones by UV-visible spectrophotometer especially by a microplate reader.

This novel microplate UV-visible approach not only made up for the imperfection of HPLC, which was not suitable for high-throughput screening and time-consuming, but also overcame the disadvantages that synthetic substrate *p*NPR could not affect the hydrolytic capacities of the α-L-rhamnosidases on natural flavonoid glycosides. Accordingly, this microplate UV-visible method might be applied for quickly exploring substrate selectivities (α-1,6 or α-1,2) of a series of mesophilic α-L-rhamnosidases on citrus flavanone diglycosides by combining them with a high-active and thermophilic β-D-glucosidase. UV-visible spectrophotometry has been used for evaluating relative activities of the α-L-rhamnosidases on rutin and high-throughput screening of the mutants library for site-directed saturation mutagenesis of the α-L-rhamnosidase in previous study [47]. However, only the rutin were applied for this method. Based on spectral difference between hesperidin and its aglycone hesperetin in pH 10.0, the spectrophotometric method has been reported for quantifying the hesperidin in citrus-based foods by using a fungal diglycosidase [46]. However, this method is only suitable for the hesperidin and the diglycosidase, and not compatible with the α-L-rhamnosidase and other citrus flavanone diglycosides.

By analyzing the molecular structures of common dietary flavonoid glycosides (Figure 1), the key affecting substrate selectivities of the α-L-rhamnosidases from human gut bacteria was the disaccharide group (neohesperidose or rutinose), that is, the glycosidic linkage (α-1,6 or α-1,2) between α-L-rhamnose group and β-D-glucose group. Furthermore, the glycosylation at different positions (7-OH or 3-OH) of the aglycones also produced a certain effect on substrate selectivities of the α-L-rhamnosidases from human gut bacteria. However, structural differences in the aglycones including the subgroups and methylation had a weak impact on substrate selectivities of the α-L-rhamnosidases from human gut bacteria.

α-L-Rhamnosidases from human gut bacteria play a key role in the metabolism and biodegradation of dietary flavonoid glycosides. The α-L-rhamnosidases from human gut bacteria possess diverse substrate selectivity on dietary flavonoid diglycosides. The differences in substrate selectivities of the α-L-rhamnosidases from human gut bacteria on dietary flavonoid glycosides will lead to individual variations in the absorption and utilization of dietary flavonoid glycosides.

## 4. Materials and Methods

### 4.1. Bacterial Strains and Reagents

The flavonoids were purchased from Yuanye Biotech (Shanghai, China) and Aladdin Scientific Corp. (Shanghai, China). Chromatographic grade acetonitrile and methanol were obtained from Merck (Darmstadt, Germany). *E. coli* BL21(DE3) was used as the host for protein over-expression. Other chemicals for buffer and medium preparation were of analytical grade or higher.

### 4.2. UV-Visible Spectra of Citrus Flavanones

Citrus flavanones (20 mM) were solubilized in dimethyl sulfoxide (DMSO) and diluted into 0.5 mM with methanol. The spectrophotometric assays were performed by adding 300 μL citrus flavanones (0.5 mM) into 700 μL of methanol, 700 μL of 50 mM NaH_2_PO_4_-Na_2_HPO_4_ buffer (pH 6.5), or 700 μL of 50 mM Na_2_CO_3_ (pH 10.0). After incubating for 30 min at room temperature, 400 μL of mixture was added into 400 μL of ddH_2_O. The spectra from 250 nm to 500 nm at 25 °C were recorded by a spectrophotometer UV-5800PC (Metash, Shanghai, China).

### 4.3. Preparation of Standard Curves of Citrus Flavanone by UV-Visible Spectroscopy Using Microplate

Standard curves for four citrus flavanone diglycosides and their corresponding aglycones were performed at the wavelength of 320 nm. A series of standard solutions consisting of citrus flavanone diglycosides and their corresponding aglycones in certain ratio (the interval of 2 mM, total concentration of 20 mM, DMSO dissolve) were prepared. 10 μL of standard solutions were mixed with 190 μL of 50 mM NaH_2_PO_4_-Na_2_HPO_4_ buffer (pH 6.5) in a 96-well microplate, and then 200 μL of methanol was added. 60 μL of the mixtures from each well in previous microplate was drawn into a UV-transparent 96-well microplate containing 140 μL of 50 mM Na_2_CO_3_ (pH 10.0) in each well. After incubating for 30 min at room temperature, the absorption values at 320 nm were measured by the microplate reader Synergy Mx (BioTek, Winooski, VT, USA).

### 4.4. Over-Expression and Purification of α-L-Rhamnosidases and β-D-Glucosidase

Four α-L-rhamnosidases from human gut bacteria and the high-active mutant TnBgl1A-DM of thermophilic β-D-glucosidase TnBgl1A from *Thermotoga neapolitana* were over-expressed in *E. coli* BL21(DE3) and were purified by Ni-NTA affinity chromatography as described in previous study [47,48]. Protein concentrations of the purified enzymes were measured at 280 nm by NanoDrop 2000 UV–visible spectrophotometer (Thermo Fisher Scientific, Waltham, MA, USA).

### 4.5. Activity Assay of α-L-Rhamnosidases by UV-Visible Spectroscopy Using Microplate

**Figure 8:**
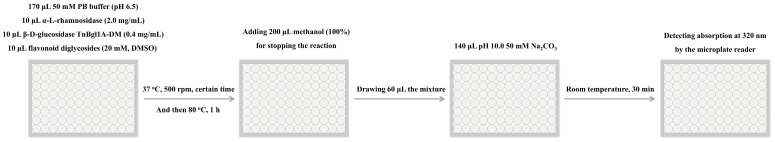
A pipeline for activity assay of α-L-rhamnosidases by UV-visible spectroscopy using microplate.

The reaction mixture of 200 μL in 96-well microplate contained 170 μL of 50 mM NaH_2_PO_4_-Na_2_HPO_4_ buffer (pH 6.5), 10 μL of citrus flavanone diglycosides (20 mM, DMSO dissolve), 10 μL of purified α-L-rhamnosidase (2.0 mg mL^−1^), and 10 μL of purified TnBgl1A-DM (0.4 mg mL^−1^). The α-L-rhamnosidase reaction was performed by shaking for varied times (0–120 min) in 500 rpm at 37 °C, and then β-D-glucosidase reaction was carried out by incubating for 1 h at 80 °C (Figure 8). The reaction was stopped by adding 200 μL of methanol. The mixture of 60 μL from each well was drawn correspondingly into each well in a UV-transparent microplate containing 140 μL of 50 mM Na_2_CO_3_ (pH 10.0). After incubating for 30 min at room temperature, the absorption values at 320 nm were measured by a microplate reader (Figure 8). Final product citrus flavanone aglycones were quantified according to standard curves (Figure S1). The conversion rate was calculated by using the following equation:

Conversion rate (%) = (aglycone concentration/initial substrate concentration) × 100.

### 4.6. Activity Assay of α-L-Rhamnosidases by HPLC

The reaction mixture of 200 μL contained 180 μL of 50 mM NaH_2_PO_4_–Na_2_HPO_4_ buffer (HFM-RhaA and HFM-RhaC, pH 6.0; BtRha78A and HFM-Rha78, pH 6.5), 10 μL of 20 mM flavonoid glycosides (dissolved in DMSO) and 10 μL of purified α-L-rhamnosidase (2.0 mg mL^−1^). After shaking for a defined time in 800 rpm at 37 °C, the reaction was stopped by adding 800 μL of methanol. After centrifuging at 13,000 rpm for 5 min, the supernatant was filtered through the 0.22 μm nylon 6 membrane filter for HPLC. Catalytic activities of the α-L-rhamnosidases were calculated by quantifying the released products or hydrolyzed substrates (corresponding product standard not yet obtained) based on the standard curves (Figure S4).

### 4.7. Enzyme Kinetics of α-L-Rhamnosidases by HPLC

Enzyme kinetics of the α-L-rhamnosidases on natural flavonoid diglycosides were determined in 50 mM NaH_2_PO_4_–Na_2_HPO_4_ buffer (HFM-RhaA and HFM-RhaC, pH 6.0; BtRha78A and HFM-Rha78, pH 6.5) at 37 °C for 5 min with a certain enzyme concentration. The initial rate of hydrolysis reaction was measured with varied concentrations of natural flavonoid diglycosides (dissolved in DMSO). Kinetics parameters V_max_ and *K*_M_ were obtained by fitting enzymatic activities at different concentrations of flavonoid diglycosides as a function of substrate concentrations into the Michaelis—Menten equation by using non-linear regression of the software GraphPad Prism 9. The parameter *k*_cat_ was calculated using the equation

*k*_cat_ = V_max_/[E]

where [E] was molar concentration of the enzymes. The molar concentration of the enzymes [E] was calculated by dividing the protein concentration measured by NanoDrop 2000 by molecular weight of the enzyme.

### 4.8. High Performance Liquid Chromatography (HPLC)

HPLC was carried out by Waters 1525 binary pump and Waters 2487 dual λ absorbance detector (Waters, Framingham, MA, USA) on a reverse-phase Hypersil OSD2-C18 column (4.6 × 150 mm, particle size 5 μm, Elite, Dalian, China) at room temperature. The flavonoid diglycosides and corresponding flavonoid glucosides were separated by using 0.5% (*v*/*v*) acetic acid (A) and acetonitrile (B) with a certain ratios (Table S1) as the mobile phase at a flow rate of 1.0 mL min^−1^ and detected at a specific wavelength (Table S1).

### 4.9. Molecular Docking of α-L-Rhamnosidases with Flavonoid Glycosides and Structural Analysis

Molecular docking of the α-L-rhamnosidases BtRha78A and HFM-RhaA with natural flavonoid glycosides was performed by the software Autodock vina 1.2.0 [49]. The grid center of BtRha78A was the position of Tris. The size of the grid box was 22.5 Å × 22.5 Å × 22.5 Å. The grid center of HFM-RhaA was the center of general acid Glu448 and general base Glu715. The size of the grid box was 18.75 Å × 18.75 Å × 18.75 Å. After 100 docking times, 50 ligand poses were evaluated and the optimal docking conformation was obtained. The docking results were analyzed and displayed by the softwares LigPlot^+^ 1.4 [50] and PyMOL 3.0.5 education [51].

## 5. Conclusions

In this work, a novel microplate spectrophotometric approach has been developed to rapidly evaluate conversion rates and substrate selectivities (α-1,6 or α-1,2) of mesophilic α-L-rhamnosidases towards citrus flavanone diglycosides by combining with a high-active and thermophilic β-D-glucosidase. Furthermore, catalytic activities and enzyme kinetics of four α-L-rhamnosidases from human gut bacteria on dietary flavonoid glycosides indicated that the α-L-rhamnosidase BtRha78A specifically removed the rhamnose group from the flavones, flavanones and flavonols diglycosides with the α-1,6 glycosidic bond. HFM-RhaA preferred to catalyze the flavones, flavanones and dihydrochalcones diglycosides containing the α-1,2 glycosidic linkages at the 7-OH of the aglycones. However, this enzyme also showed high catalytic activity on the flavonol diglycoside rutin with the α-1,6 glycosidic bond at the 3-OH of the aglycones. HFM-RhaC exhibited certain hydrolytic abilities towards all flavonoid diglycosides, and displayed higher catalytic activities on the flavonoid diglycosides with the α-1,6 glycosidic bonds. HFM-Rha78 weakly hydrolyzed the flavones, flavanones and dihydrochalcones diglycosides with the α-1,2 glycosidic bonds, and the flavonols diglycosides with α-1,6 glycosidic bonds. Finally, structural analysis by molecular docking revealed that the aromatic residues Tyr383, Trp440, Tyr610 and the charged residues Arg37, Asp335, Asp342, Glu595, His620 in BtRha78A formed hydrogen bonds with catalytically active flavonoid diglycosides. The residue Ala498 in HFM-RhaA interacted with the flavonoid diglycosides naringin, rhoifolin, rutin and hesperidin by hydrogen bonds, and the residue Ala498 might be crucial for catalytic activity of this enzyme on the flavonoid diglycosides.

## Figures and Tables

**Table 1:** Substrate selectivities of reported bacterial GH78 family α-L-rhamnosidases on natural flavonoid glycosides.

Enzymes	Organism	GenBank Accession	Selectivity on Natural Flavonoid Glycosides	Ref.
RhaB	*Bacillus* sp. GL1	BAB62315	Might catalyzed naringin	[34]
BbRha	*Bifidobacterium breve* 689b	CP006715	Might hydrolyzed hesperidin, naringin and rutin	[35]
BdRham	*Bifidobacterium dentium* K-13	AGS77942	Might biotransformed naringin and rutin Could not hydrolyze quercitrin	[36]
RamA	*Clostridium stercorarium*	AJ238748	Might hydrolyzed hesperidin and naringin	[37]
DtRha	*Dictyoglomus thermophilum*	ACI19983	High activities on naringin, neodiosimin and neohesperidin Low activities on narirutin, diosimin and hesperidin	[4]
KoRha	*Klebsiella oxytoca*	YP_005019950	Might catalyzed rutin	[5]
RhaB2	*Lactobacillus plantarum* NCC245	ACR19005	Might hydrolyzed hesperidin and rutin Could not catalyze naringin and quercitrin	[38]
RhaB1	*Lactobacillus plantarum* NCC245	ACR19007	Might hydrolyzed hesperidin and rutin Could not catalyze naringin and quercitrin	[38]
Ram1	*Lactobacillus plantarum* WCFS1	CAD65558	Might hydrolyzed narirutin and rutin Low bioconversion rate on naringin	[39]
Ram2	*Pediococcus acidilactici*	WP_004165637	Might hydrolyzed hesperidin and rutin	[40]
Ram	*Pediococcus acidilactici*	EFL96112	Might biotransformed rutin Low bioconversion rate on hesperidin	[40]
SaRha78A	*Streptomyces avermitilis* MA-4680	BAC68538	Might biotransformed naringin and rutin Low bioconversion rate on hesperidin	[41]
RhmB	Thermomicrobia bacterium PRI-1686	AAR96047	Might hydrolyzed hesperidin and naringin Could not hydrolyze rutin	[42]
RhmA	Thermomicrobia bacterium PRI-1686	AAR96046	Might hydrolyzed hesperidin and naringin Slightly hydrolyzed rutin	[42]
RhaB1	Elephant feces metagenome	ZP01961192	Low bioconversion rates on naringin and rutin	[43]

**Table 2:** Hydrolytic activities of four α-L-rhamnosidases from human gut bacteria on flavonoids glycosides.

Substrates	Glycosidic Bonds	Specific Activity (U g^−1^)			
		BtRha78A	HFM-RhaA	HFM-RhaC	HFM-Rha78
Rutin	α-1,6	84.6 ± 2.0	1313.1 ± 44.9	188.6 ± 6.6	13.9 ± 0.2
Troxerutin	α-1,6	13.9 ± 1.5	312.5 ± 9.2	62.9 ± 4.6	13.2 ± 0.3
Quercitrin	α-1	NA	NA	NA	NA
Myricetrin	α-1	NA	NA	NA	NA
Icariin	α-1	NA	NA	NA	NA
Diosmin	α-1,6	2050.7 ± 14.1	NA	1886.3 ± 3.0	NA
Rhoifolin	α-1,2	NA	1184.0 ± 19.3	43.1 ± 3.5	415.0 ± 15.7
Hesperidin	α-1,6	1399.0 ± 38.4	14.3 ± 0.6	2228.9 ± 34.8	NA
Neohesperidin	α-1,2	NA	1699.6 ± 54.2	259.5 ± 6.5	88.5 ± 1.0
Methy hesperidin	α-1,6	1205.1 ± 20.0	7.9 ± 0.6	1590.1 ± 3.4	NA
Naringin	α-1,2	NA	1925.4 ± 44.7	203.8 ± 1.3	129.4 ± 1.6
Narirutin	α-1,6	2034.1 ± 43.4	26.1 ± 0.8	2361.5 ± 56.4	NA
Neohesperidin dihydrochalcone	α-1,2	NA	1464.6 ± 5.4	86.6 ± 0.4	24.7 ± 0.7
Naringin dihydrochalcone	α-1,2	NA	1258.6 ± 26.6	60.5 ± 1.5	25.3 ± 2.0

One unit of enzyme activities (U) on the substrates rutin, hesperidin, neohesperidin, naringin and narirutin were defined as the amount of enzyme releasing 1 μmol the product at 37 °C in pH 6.5 or 6.0. One unit of enzyme activities (U) on the substrates troxerutin, diosmin, rhoifolin, methy hesperidin, neohesperidin dihydrochalcone and naringin dihydrochalcone were defined as the amount of enzyme hydrolyzing 1 μmol the substrate per min at 37 °C in pH 6.5 or 6.0. NA represents no activity detected. All reactions were performed in triplicate. The specific activities represent the mean ± standard deviation.

**Table 3:** Enzyme kinetic parameters of four α-L-rhamnosidases from human gut bacteria on four dietary flavonoids diglycosides.

	BtRha78A			HFM-RhaA			HFM-RhaC			HFM-Rha78		
Substrates	*k*_cat_ (s^−1^)	*K*_M_ (mM)	*k*_cat_/*K*_M_ (s^−1^ M^−1^)	*k*_cat_ (s^−1^)	*K*_M_ (mM)	*k*_cat_/*K*_M_ (s^−1^ M^−1^)	*k*_cat_ (s^−1^)	*K*_M_ (mM)	*k*_cat_/*K*_M_ (s^−1^ M^−1^)	*k*_cat_ (s^−1^)	*K*_M_ (mM)	*k*_cat_/*K*_M_ (s^−1^ M^−1^)
Rutin	0.47	2.38	199.5	5.62	1.24	4546.6	2.31	5.05	457.9	ND	ND	ND
Hesperidin	8.73	2.78	3138.8	ND	ND	ND	58.38	2.16	27,025.0	ND	ND	ND
Neohesperidin	ND	ND	ND	5.39	0.53	10,154.0	3.33	3.25	1025.8	0.56	3.97	140.0
Naringin	ND	ND	ND	25.91	1.92	13,512.0	1.50	2.29	654.2	1.44	5.97	240.6

ND indicates not determined.

## Data Availability

Data are contained within the article and Supplementary Materials.

## Supplementary Materials

The following supporting information can be downloaded at: https://www.mdpi.com/article/10.3390/molecules30050980/s1, Table S1: HPLC conditions for dietary flavonoid glycosides; Figure S1: Standard curves for quantification of citrus flavanone diglycosides and their corresponding aglycones using microplate reader at 320 nm in pH 10.0; Figure S2: SDS-PAGE (15%) for purification of four mesophilic α-L-rhamnosidases from human gut bacteria; Figure S3: SDS-PAGE (15%) for purification of high-active mutant TnBgl1A-DM of thermophilic β-D-glucosidase TnBgl1A from *Thermotoga neapolitana*; Figure S4: Standard curves for quantification of flavonoid glycosides by HPLC.

## References

[1] Yadav V., Yadav P.K., Yada S., Yadav K.D.S. (2010). α-L-Rhamnosidase: A review. Process Biochem..

[2] Cui Z., Maruyama Y., Mikami B., Hashimoto W., Murata K. (2007). Crystal structure of glycoside hydrolase family 78 alpha-L-rhamnosidase from *Bacillus* sp. GL1. J. Mol. Biol..

[3] Bonanno J.B., Almo S.C., Bresnick A. (2005). New York-Structural GenomiX Research Consortium (NYSGXRC): A large scale center for the protein structure initiative. J. Struct. Funct. Genom..

[4] Guillotin L., Kim H., Traore Y., Moreau P., Lafite P., Coquoin V., Nuccio S., de Vaumas R., Daniellou R. (2019). Biochemical characterization of the α-L-rhamnosidase DtRha from *Dictyoglomus thermophilum*: Application to the selective derhamnosylation of natural flavonoids. ACS Omega.

[5] O’Neill E.C., Stevenson C.E., Paterson M.J. (2015). Crystal structure of a novel two domain GH78 family α-rhamnosidase from *Klebsiella oxytoca* with rhamnose bound. Proteins.

[6] Fujimoto Z., Jackson A., Michikawa M. (2013). The structure of a *Streptomyces avermitilis* α-L-rhamnosidase reveals a novel carbohydrate-binding module CBM67 within the six-domain arrangement. J. Biol. Chem..

[7] Berman H.M., Westbrook J., Feng Z., Gilliland G., Bhat T.N., Weissig H., Shindyalov I.N., Bourne P.E. (2000). The Protein Data Bank. Nucleic Acids Res..

[8] Makabe K., Ishida N., Kanezaki N., Shiono Y., Koseki T. (2024). *Aspergillus oryzae* α-L-rhamnosidase: Crystal structure and insight into the substrate specificity. Proteins.

[9] Pachl P., Škerlová J., Šimčíková D. (2018). Crystal structure of native α-L-rhamnosidase from *Aspergillus terreus*. Acta Crystallogr. D Struct. Biol..

[10] Li L.J., Peng C., Gong J.Y., Liu X.Q., Li W.J., Zhu Y.B., Ni H., Li Q.B. (2023). Improving alkaline stability of α-L-rhamnosidase from *Aspergillus niger* through computational strategy combines with folding free energy and binding free energy. Biochem. Eng. J..

[11] Li L.J., Liu X.Q., Du X.P., Wu L., Jiang Z.D., Ni H., Li Q.B., Chen F. (2020). Preparation of isoquercitrin by biotransformation of rutin using α-L-rhamnosidase from *Aspergillus niger* JMU-TS528 and HSCCC purification. Prep. Biochem. Biotechnol..

[12] Céliz G., Rodriguez J., Soria F., Daz M. (2015). Synthesis of hesperetin 7-O-glucoside from flavonoids extracted from citrus waste using both free and immobilized α-L-rhamnosidases. Biocatal. Agric. Biotechnol..

[13] Luo C.M., Ke L.F., Huang X.Y., Zhuang X.Y., Xiao A.F., Zhang Y.H. (2024). Efficient biosynthesis of prunin in methanol cosolvent system by an organic solvent-tolerant α-L-rhamnosidase from *Spirochaeta thermophila*. Enzyme Microb. Technol..

[14] Li L.J., Wu Z.Y., Yu Y., Zhang L.J., Zhu Y.B., Ni H., Chen F. (2018). Development and characterization of an α-L-rhamnosidase mutant with improved thermostability and a higher efficiency for debittering orange juice. Food Chem..

[15] Spagma G., Barbagallo R.N., Martino A., Pifferi P.G. (2000). A simple method of purifying glycosidase: α-L-rhamnopyranosidases from *Aspergillus niger* to increase the aroma of Moscato wine. Enzyme Microb. Technol..

[16] Ni H., Xiao A.F., Wang Y.Q., Chen F., Cai H.N., Su W.J. (2013). Development and evaluation of an HPLC method for accurate determinations of enzyme activities of naringinase complex. J. Agric. Food Chem..

[17] Monti D., Pisvejcová A., Kren V., Lama M., Riva S. (2004). Generation of an α-L-rhamnosidase library and its application for the selective derhamnosylation of natural products. Biotechnol. Bioeng..

[18] Xie J.C., Zhao J., Zhang N., Xu H., Yang J., Ye J., Jiang J.C. (2022). Efficient production of isoquercitin, icariin and icariside II by a novel thermostable α-L-rhamnosidase PodoRha from *Paenibacillus odorifer* with high α-1, 6-/α-1, 2-glycoside specificity. Enzym. Microb. Technol..

[19] Tripoli E., Guardia L.M., Giammanco S., Di Majo D., Giammanco M. (2006). Citrus flavonoids: Molecular structure, biological activity and nutritional properties: A review. Food Chem..

[20] Ciftci O., Ozcan C., Kamisli O., Cetin A., Basak N., Aytac B. (2015). Hesperidin, a citrus flavonoid, Has the ameliorative effects against experimental autoimmune encephalomyelitis (EAE) in a C57BL/J6 mouse model. Neurochem. Res..

[21] Wu F., Lei H.H., Chen G., Chen C., Song Y., Cao Z., Zhang C., Zhang C., Zhou J., Lu Y. (2021). In vitro and in vivo studies reveal that hesperetin-7-O-glucoside, a naturally occurring monoglucoside, exhibits strong anti-inflammatory capacity. J. Agri. Food Chem..

[22] Ramanan M., Sinha S., Sudarshan K., Aidhen I.S., Doble M. (2016). Inhibition of the enzymes in the leukotriene and prostaglandin pathways in inflammation by 3-aryl isocoumarins. Eur. J. Med. Chem..

[23] Pandey P., Khan F. (2021). A mechanistic review of the anticancer potential of hesperidin, a natural flavonoid from citrus fruits. Nutr. Res..

[24] Tutunchi H., Naeini F., Ostadrahimi A., Hosseinzadeh-Attar M.J. (2020). Naringenin, a flavanone with antiviral and anti-inflammatory effects: A promising treatment strategy against COVID-19. Phytother. Res..

[25] Gandhi G.R., Vasconcelos A.B.S., Wu D.T., Li H.B., Antony P.J., Li H., Geng F., Gurgel R.Q., Narain N., Gan R.Y. (2020). Citrus flavonoids as promising phytochemicals targeting diabetes and related complications: A systematic review of in vitro and in vivo studies. Nutrients.

[26] Hwang S.L., Shih P.H., Yen G.C. (2012). Neuroprotective effects of citrus flavonoids. J. Agric. Food Chem..

[27] Sudarshan K., Boda A.K., Dogra S., Bose I., Yadav P.N., Aidhen I.S. (2019). Discovery of an isocoumarin analogue that modulates neuronal functions via neurotrophin receptor TrkB. Bioorg. Med. Chem. Lett..

[28] Mahmoud A.M., Hernández Bautista R.J., Sandhu M.A., Hussein O.E. (2019). Beneficial effects of citrus flavonoids on cardiovascular and metabolic health. Oxid. Med. Cell Longev..

[29] Slámová K., Kapešová J., Valentová K. (2018). “Sweet flavonoids”: Glycosidase-catalyzed modifications. Int. J. Mol. Sci..

[30] Chang H.Y., Lee Y.B., Bae H.A., Huh J.Y., Nam S.H., Sohn S.H., Lee H.J., Lee S.B. (2011). Purification and characterisation of *Aspergillus sojae* naringinase: The production of prunin exhibiting markedly enhanced solubility with in vitro inhibition of HMG-CoA reductase. Food Chem..

[31] Lee Y.S., Huh J.Y., Nam S.H., Moon S.K., Lee S.B. (2012). Enzymatic bioconversion of citrus hesperidin by *Aspergillus sojae* naringinase: Enhanced solubility of hesperetin-7-O-glucoside with in vitro inhibition of human intestinal maltase, HMG-CoA reductase, and growth of *Helicobacter pylori*. Food Chem..

[32] Park H.Y., Choi H.D., Eom H., Choi I. (2013). Enzymatic modification enhances the protective activity of citrus flavonoids against alcohol-induced liver disease. Food Chem..

[33] Liu A., Huang B., Lei L., Lu Y.J., Zhou J.L., Wong W.L. (2019). Production of high antioxidant activity flavonoid monoglucosides from citrus flavanone with immobilised α-L-rhamnosidase in one step. Int. J. Food Sci. Technol..

[34] Hashimoto W., Miyake O., Nankai H., Murata K. (2003). Molecular identification of an α-L-rhamnosidase from *Bacillus* sp. strain GL1 as an enzyme involved in complete metabolism of gellan. Arch. Biochem. Biophys..

[35] Zhang R., Zhang B.L., Xie T., Li G.C., Tuo Y., Xiang Y.T. (2015). Biotransformation of rutin to isoquercitrin using recombinant α-L-rhamnosidase from *Bifidobacterium breve*. Biotechnol. Lett..

[36] Bang S.H., Hyun Y.J., Shim J., Hong S.W., Kim D.H. (2015). Metabolism of rutin and poncirin by human intestinal microbiota and cloning of their metabolizing α-L-rhamnosidase from *Bifidobacterium dentium*. J. Microbiol. Biotechnol..

[37] Zverlov V.V., Hertel C., Bronnenmeier K., Hroch A., Kellermann J., Schwarz W.H. (2000). The thermostable α-L-rhamnosidase RamA of *Clostridium stercorarium*: Biochemical characterization and primary structure of a bacterial α-L-rhamnoside hydrolase, a new type of inverting glycoside hydrolase. Mol. Microbiol..

[38] Ávila M., Jaquet M., Moine D., Requena T., Peláez C., Arigoni F., Jankovic I. (2009). Physiological and biochemical characterization of the two α-L-rhamnosidases of *Lactobacillus plantarum* NCC245. Microbiology.

[39] Beekwilder J., Marcozzi D., Vecchi S., de Vos R., Janssen P., Francke C., van Hylckama Vlieg J., Hall R.D. (2009). Characterization of rhamnosidases from *Lactobacillus plantarum* and *Lactobacillus acidophilus*. Appl. Environ. Microbiol..

[40] Michlmayr H., Brandes W., Eder R., Schümann C., del Hierro A.M., Kulbe K.D. (2011). Characterization of two distinct glycosyl hydrolase family 78 α-L-rhamnosidases from *Pediococcus acidilactici*. Appl. Environ. Microbiol..

[41] Ichinose H., Fujimoto Z., Kaneko S. (2013). Characterization of an α-L-rhamnosidase from *Streptomyces avermitilis*. Biosci. Biotechnol. Biochem..

[42] Hákon B., Gudmundur O.H., Olafur H.F., Andrew M., Jakob K.K., Bo M. (2004). Two new thermostable α-L-rhamnosidases from a novel thermophilic bacterium. Enzym. Microb. Technol..

[43] Rabausch U., Ilmberger N., Streit W.R. (2014). The metagenome-derived enzyme RhaB opens a new subclass of bacterial B type α-L-rhamnosidases. J. Biotechnol..

[44] Weiz G., Breccia J.D., Mazzaferro L.S. (2017). Screening and quantification of the enzymatic deglycosylation of the plant flavonoid rutin by UV-visible spectrometry. Food Chem..

[45] Khan S., Pozzo T., Megyeri M., Lindahl S., Sundin A., Turner C., Karlsson E.N. (2011). Aglycone specificity of *Thermotoga neapolitana* β-glucosidase 1A modified by mutagenesis, leading to increased catalytic efficiency in quercetin-3-glucoside hydrolysis. BMC Biochem..

[46] Mazzaferro L.S., Breccia J.D. (2012). Quantification of hesperidin in citrus-based foods using a fungal diglycosidase. Food Chem..

[47] Li B.C., Peng B., Zhang T., Li Y.Q., Ding G.B. (2019). A spectrophotometric method for high-throughput screening of α-L-rhamnosidase activity on rutin coupled with a β-D-glucosidase assay. 3 Biotech.

[48] Zhang T., Li Y.Q., Ding G.B. (2019). Target discovery of novel α-L-rhamnosidases from human fecal netagenome and application for biotransformation of natural flavonoid glycosides. Appl. Biochem. Biotechnol..

[49] Eberhardt J., Santos-Martins D., Tillack A.F., Forli S. (2021). AutoDock Vina 1.2.0: New docking methods, expanded force field, and python bindings. J. Chem. Inf. Model..

[50] Laskowski R.A., Swindells M.B. (2011). LigPlot+: Multiple ligand-protein interaction diagrams for drug discovery. J. Chem. Inf. Model..

[51] (2024). The PyMOL Molecular Graphics System.

