# Supplementary material for: Substrate Selectivities of GH78 α-L-Rhamnosidases from Human Gut Bacteria on Dietary Flavonoid Glycosides
Source: Molecules. 2025 Feb 20;30(5):980. doi: 10.3390/molecules30050980 (PMC11901676; doi:10.3390/molecules30050980)
Supplement: Supplementary file 1 [file molecules-30-00980-s001.zip › molecules-3484368-supplementary.pdf]

Supplementary Material

Substrate Selectivities of GH78 α-L-Rhamnosidases from Human Gut Bacteria on Dietary Flavonoid Glycosides

Table S1. HPLC conditions for dietary flavonoid glycosides

Flavonoid glycosides	Detection wavelength (nm)	Mobile phase 0.5% (v/v) Acetic acid (A) : Acetonitrile (B)
Rutin	260	84 : 16
Troxerutin	260	82 : 18
Quercitrin	260	77 : 23
Myricetrin	260	78 : 22
Icariin	270	67 : 33
Diosmin	283	77 : 23
Rhoifolin	340	78 : 22
Hesperidin	283	77 : 23
Neohesperidin	283	77 : 23
Methyl hesperidin	283	77 : 23
Naringin	283	77 : 23
Narirutin	283	77 : 23
Neohesperidin dihydrochalcone	283	74 : 26
Naringin dihydrochalcone	283	74 : 26

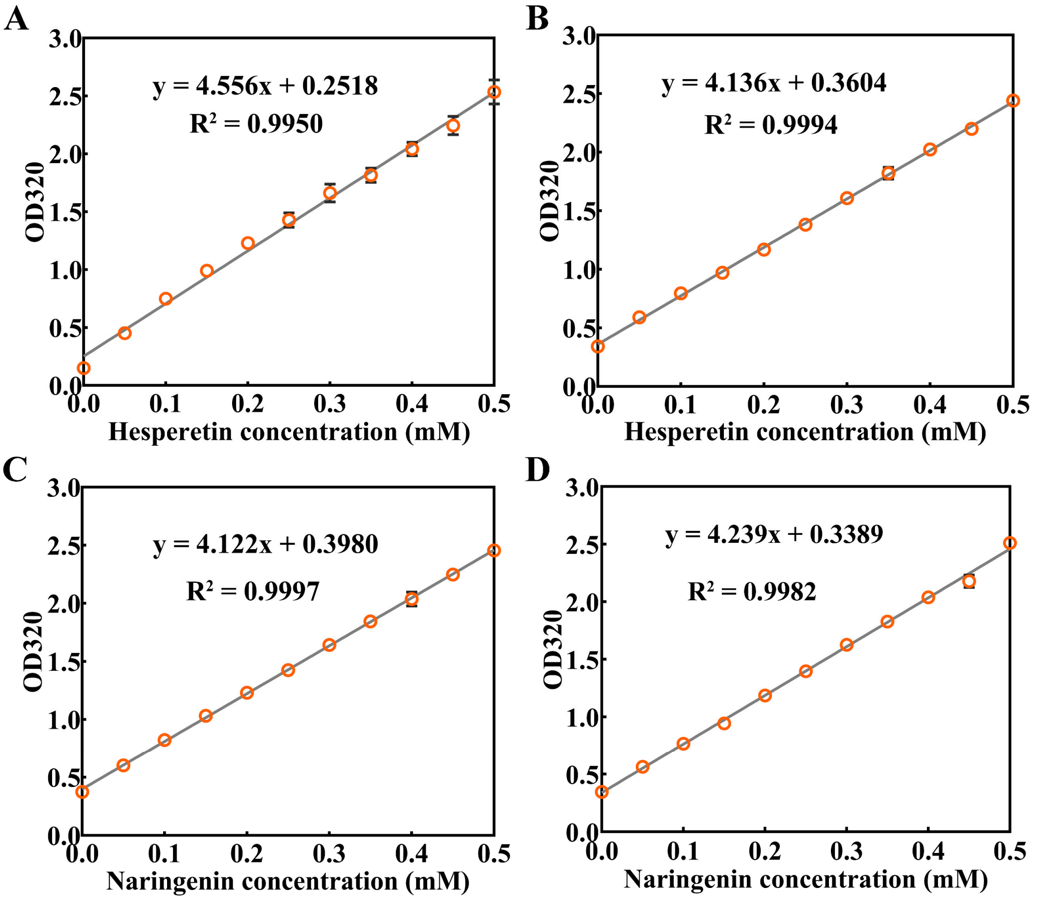

Figure S1. Standard curves for quantification of citrus flavanone diglycosides and their corresponding aglycones at 320 nm in pH 10.0. (A) Standard curve for hesperidin and hesperetin, (B) Standard curve for neohesperidin and hesperetin, (C) Standard curve for naringin and naringenin, (D) Standard curve for narirutin and naringenin. All reactions were performed in triplicate, and error bars represent the standard deviations of mean.

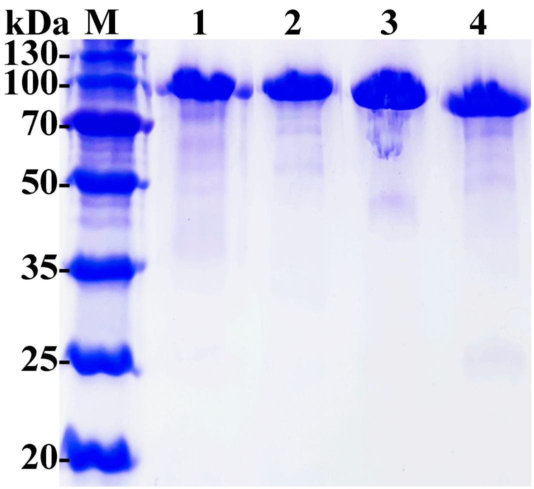

Figure S2. SDS-PAGE (15%) for purification of four mesophilic α-L-rhamnosidases from human gut bacteria. Lane M represents the protein standard marker, lanes 1-4 represent the purified HFM-RhaA, HFM-RhaC, HFM-Rha78, and BtRha78A, respectively.

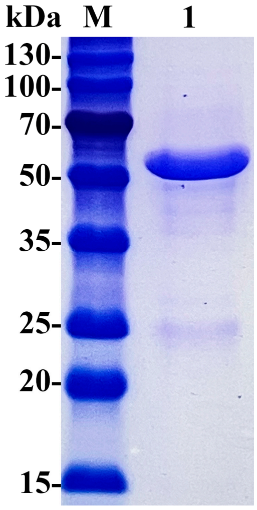

Figure S3. SDS-PAGE (15%) for purification of high-active mutant TnBgl1A-DM of thermophilic β-D-glucosidase TnBgl1A from *Thermotoga neapolitana*. Lane M represents the protein standard marker, lane 1 represents the purified TnBgl1A-DM.

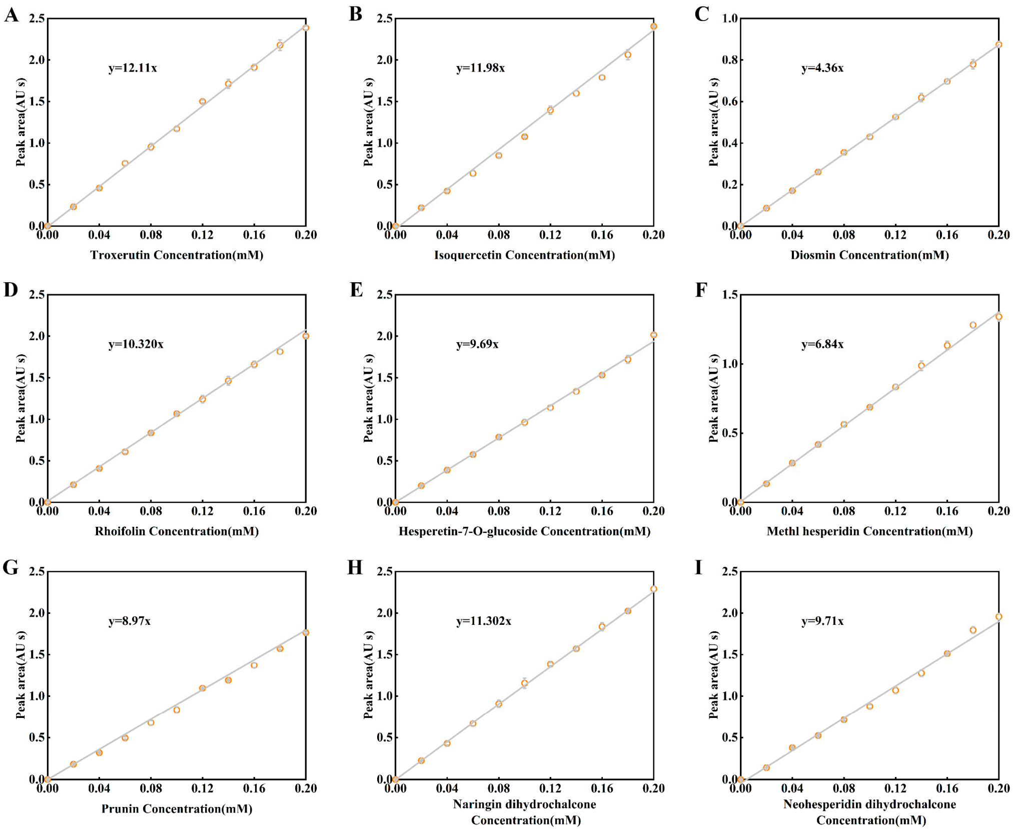

Figure S4. Standard curves for quantification of flavonoid glycosides by HPLC. (A) Standard curve of troxerutin, (B) Standard curve of isoquercetin, (C) Standard curve of diosmin, (D) Standard curve of rhoifolin, (E) Standard curve of hesperetin-7-O-glucoside, (F) Standard curve of methyl hesperidin, (G) Standard curve of prunin, (H) Standard curve of naringin dihydrochalcone, (I) Standard curve of neohesperidin dihydrochalcone. All reactions were performed in triplicate, and error bars represent the standard deviations of mean.
